# Acute parkinsonism in a patient with myxedema crisis: A case report

**DOI:** 10.3892/mi.2024.173

**Published:** 2024-07-02

**Authors:** Rajat Gupta, Shakun Chaudhary, Vivek Sood, Narvir Singh Chauhan, Nidhi Chauhan, Dhiraj Kapoor

**Affiliations:** 1 Department of Medicine, Dr Rajendra Prasad Government Medical College Kangra at Tanda, Kangra, Himachal Pradesh 176001, India; 2 Department of Endocrinology, Dr Rajendra Prasad Government Medical College Kangra at Tanda, Kangra, Himachal Pradesh 176001, India; 3 Department of Radiology, Dr Rajendra Prasad Government Medical College Kangra at Tanda, Kangra, Himachal Pradesh 176001, India

**Keywords:** acute parkinsonism, myxedema crisis, Sheehan's syndrome

## Abstract

Both myxedema crisis and Sheehan's syndrome are uncommon conditions. The first-time presentation as myxedema crisis is rare in Sheehan's syndrome. The present study describes the case of a 31-year-old female patient who presented with altered sensorium in the emergency room. The patient was not a known case of hypothyroidism, but had a history of secondary amenorrhea and lactation failure following the birth of a child 11 years prior. Upon evaluation, she was found to have hypothermia, hypotension, the delayed relaxation of deep tendon reflexes, bradycardia and hyponatremia, which led to the suspicion of myxedema crisis. Her thyroid function tests were suggestive of secondary hypothyroidism and her pituitary hormonal profile revealed panhypopituitarism. The patient was managed on the lines of myxedema crisis with oral levothyroxine, hydrocortisone infusion, antibiotics and rewarming. Her clinical and biochemical parameters exhibited an improvement; however, her altered sensorium persisted. A repeat neurological examination revealed cogwheel rigidity with paraparesis, which led to the clinical suspicion of acute parkinsonism. Magnetic resonance imaging of the sella and brain was suggestive of an empty sella and extrapontine myelinolysis, substantiating the diagnosis of Sheehan's syndrome with acute parkinsonism. The patient was commenced on levodopa-carbidopa following which there was an improvement in symptoms. The patient improved over the ensuing 6 months and can now perform all household activities. On the whole, the present study indicates that the early suspicion of myxedema crisis, prompt treatment and the recognition of additional aetiology for persistent altered sensorium can result in a successful outcome for the patient.

## Introduction

Myxedema crisis is a decompensated form of hypothyroidism associated with high mortality rates. The condition is more common among females and the elderly. A notable proportion of patients (39-51%) are diagnosed with hypothyroidism at their presentation as myxedema crisis (1,2). Central hypothyroidism accounts for 5-17% of patients presenting with myxedema crisis (2,3). Sheehan's syndrome is a rare condition characterized by panhypopituitarism due to post-partum pituitary necrosis. Sheehan's syndrome presenting as a myxedema crisis is extremely rare.

The present study describes the case of a patient with Sheehan's syndrome with a first-time presentation as myxedema crisis. Furthermore, the patient developed acute parkinsonism during the recovery phase of the myxedema crisis, resulting in a prolongation of the altered sensorium. Acute parkinsonism leading to prolongation of the altered sensorium in a patient of myxedema crisis has not been reported previously, at least to the best of our knowledge.

## Case report

A 31-year-old female patient presented with multiple episodes of vomiting for 1 week and hypoactive delirium for 3 days. The patient was evaluated at a local medical facility for the same and was found to have hyponatremia. She was managed for hyponatremia with 3% saline, but was referred to the Tertiary Care Centre at Dr Rajendra Prasad Government Medical College Kangra at Tanda (Kangra, India) due to the persistence of altered sensorium. Upon evaluation, she was found to have a history of fever 1 week prior. She had no history of headaches, abnormal body movements, or weakness in any body part. Her previous history was significant, with secondary amenorrhea and the failure of lactation following the delivery of a child 11 years prior. The child was delivered at home and there was no history of post-partum haemorrhage. At the time of admission, the patient was drowsy with a Glasgow coma scale of 11/15, had bradycardia (pulse rate-, 50 beats/min) and hypotension (blood pressure, 84/50 mmHg). Her oral temperature was 91.5˚F (33.1˚C) and she had pallor. Upon a neurological examination, the pupils were found to be bilaterally equal and reacting to light. There was no facial asymmetry. Neck rigidity and Kernig's sign were absent. There was a delayed relaxation of deep tendon reflexes. Bilateral plantar reflexes were flexor. A cardiovascular examination revealed bradycardia, although other systemic examinations were within normal limits.

**Figure 1:**
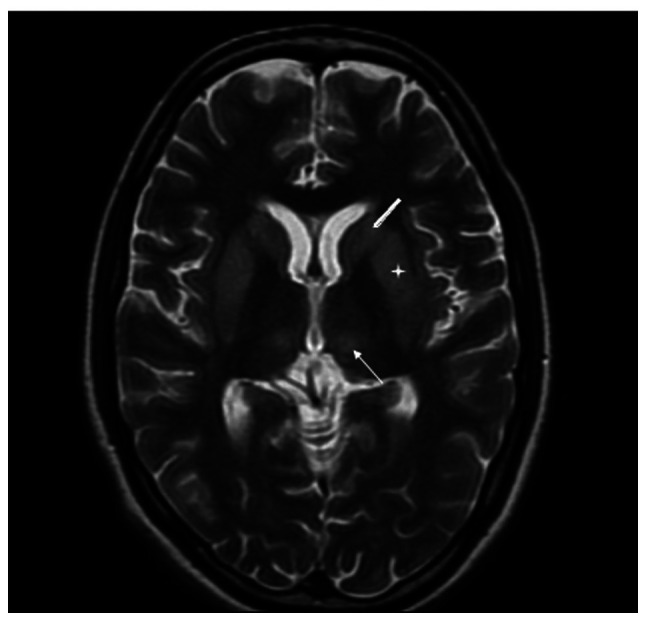
Magnetic resonance imaging of the brain (axial view; T2W) illustrating hyperintensity in bilateral caudate nuclei (thick arrow), putamen (asterisk) and thalamus (thin arrow).

**Figure 2:**
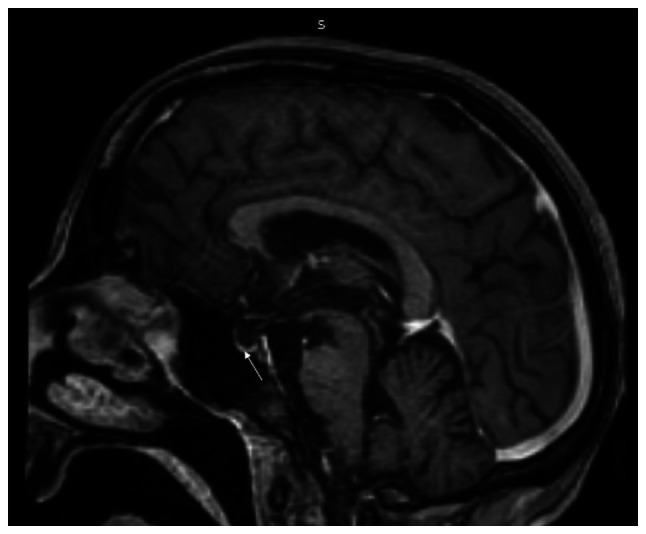
Magnetic resonance imaging (sagittal view; T1W with contrast) illustrating an empty sella (arrow).

An analysis revealed that her haemoglobin level was 106 gm/l (normal range, 120-150 gm/l), the total leucocyte count was 4.1x10^9^/l (normal range, 4-10) and her platelet count was 90,000/mm³ (range, 1.5-4.5 lakh); the differential counts were: Polymorphs, 88%; lymphocytes, 10%; eosinophils, 1%; monocytes, 1%; erythrocyte sedimentation rate, 40 mm/1st hour; random blood glucose, 4.662 mmol/l; urea, 7.14 mmol/l (normal range, 4.64-16.06 mmol/l); creatinine, 33.5 µmol/l (normal range, 35.36-106.08 µmol/l); serum sodium, 120 mmol/l (normal range, 135-155 mmol/l); serum potassium, 4 mmol/l (normal range, 3.5-5.5 mmol/l); aspartate aminotransferase, 56 IU (normal range, 5-34 IU); alanine aminotransferase, 53 IU (normal range, 6-40 IU); and alkaline phosphatase, 73 IU (normal range, 15-112 IU). Scrub and dengue serology were negative. HBsAg, hepatitis C virus and HIV serology were non-reactive. The results of a urine examination, chest X-ray and ultrasound abdomen were all within normal limits. Her arterial blood gas analysis, cerebrospinal fluid examination and electroencephalogram were within normal limits. Given her history of altered sensorium, the delayed relaxation of deep tendon reflexes, bradycardia and hyponatremia, myxedema crisis was suspected and her myxedema score was calculated. Her myxedema score was 75, suggesting myxedema crisis. Thyroid function tests revealed that T3 was <0.616 nmol/l (normal range, 1.08-3.14 nmol/l), T4 was 39.12 nmol/l (normal range, 64.35-141.57 nmol/l) and thyroid stimulating hormone (TSH) was 1.14 mIU/l (normal range, 0.550-4.780 mIU/l), suggestive of secondary hypothyroidism as opposed to sick euthyroid syndrome. Since there was a history of lactation failure, secondary amenorrhea and the delayed relaxation of deep tendon reflexes, the possibility of secondary hypothyroidism was kept. Samples for the pituitary hormonal profile were sent and management for the myxedema crisis was initiated. The patient was managed with oral levothyroxine 500 µg stat followed by 100 µg once daily through a Ryle's tube, hydrocortisone infusion, intravenous fluids, injectable antibiotics and rewarming. Her pituitary hormonal profile was suggestive of panhypopituitarism [prolactin, 76.08 pmol/l (normal range, 145.21-1,161.73 pmol/l); adrenocorticotropic hormone, 1.30 pmol/l (normal range, 1.58-13.92 pmol/l); cortisol, 108.98 nmol/l (normal range, 120.18-626.75 nmol/l); luteinising hormone, 5.4 IU/l (normal range, 1.9-12.5 IU/l); follicle stimulating hormone, 3.11 IU/l (normal range, 3.85-8.78 IU/l); and estradiol, 36.71 pmol/l (normal range, 71.58-528.62 pmol/l)]. The hormonal profile of the patient is presented in Table I. The patient exhibited an improvement in bradycardia, hypotension and hyponatremia over the ensuing 4 days; however, there was no improvement in the altered sensorium. She was awake, but not able to speak or move her limbs. A repeat neurological examination was performed, which revealed cogwheel rigidity and paraparesis. The possibility of acute parkinsonism was kept. Magnetic resonance imaging of the sella and brain was suggestive of an empty sella and extrapontine myelinolysis (Figs. 1 and 2), substantiating the diagnosis of Sheehan's syndrome with acute parkinsonism. The patient was commenced on levodopa/carbidopa following which there was a partial improvement in symptoms. She was discharged on oral hydrocortisone, levothyroxine, ethinyl estradiol and progesterone, and physiotherapy was recommended. At the 6th month of follow-up, the patient was communicating well, the paraparesis had improved and she could perform all household activities.

## Discussion

Sheehan's syndrome is characterised by the development of hypopituitarism due to post-partum pituitary necrosis. Severe post-partum haemorrhage leads to an impairment in the blood supply of the physiologically enlarged pituitary gland during pregnancy, culminating in pituitary necrosis. Owing to the improvement in obstetrical care, Sheehan's syndrome is currently rare in developed countries, but is still frequently encountered in developing nations. In addition, it is more frequently encountered with home deliveries as compared to institutional deliveries (4). Although post-partum haemorrhage is the most critical factor determining the development of Sheehan's syndrome, other factors, such as genetic susceptibility and autoimmunity also play a role. Growth hormone deficiency is the most common deficiency observed in different studies (5-8). However, data regarding prolactin and TSH deficiencies are variable. Although previous studies have reported prolactin deficiency to be more frequent, a study in India observed TSH deficiency to be more common (9). Chronic Sheehan's syndrome typically has non-specific features such as easy fatigability, lassitude, giddiness, etc., and thus often remains undiagnosed for years. The first-time presentation of Sheehan's syndrome in the emergency room as a myxedema crisis is rare. In a previous study with a cohort of 23 patients of myxedema crisis, 3 patients had Sheehan's syndrome as the aetiology of hypothyroidism (1).

Myxedema crisis is an under-recognised differential of patients visiting the emergency department with an altered sensorium. The most critical part of the management of a myxedema crisis is the timely suspicion of diagnosis and early initiation of treatment (2). Hyponatremia is a common metabolic abnormality in the emergency room and can itself present as an altered sensorium. Hence, the suspicion of myxedema crisis in the setting of hyponatremia, particularly when the patient has not previously been diagnosed with hypothyroidism, becomes difficult. The absence of features of frank hypothyroidism in secondary hypothyroidism due to TSH-independent secretion of levothyroxine, further adds to the difficulty (10). Moreover, the thyroid profile in secondary hypothyroidism is similar to sick euthyroid syndrome, which can be seen in critically ill patients of any aetiology. The history of lactation failure and secondary amenorrhea in the patient described herein was the key to the suspicion of secondary hypothyroidism. The presence of a multitude of features like hyponatremia, hypotension, bradycardia, hypothermia, etc. in a patient of altered sensorium indicates towards the diagnosis of myxedema crisis in such a setting. The use of myxedema score >60 aids in the confirmation of diagnosis (11).

Extrapontine myelinolysis (EPM) as a cause of persistent altered sensorium during the recovery phase of myxedema crisis has not been reported previously, at least to the best of our knowledge. Persistent altered sensorium in a patient with myxedema crisis can be due to electrolyte abnormalities, cerebrovascular accident, septic encephalopathy, myxedema psychosis and slow recovery in the elderly. Extrapontine myelinolysis occurs due to a rapid osmotic shift and usually develops 7-14 days following an acute insult (12). Alcoholism, malnutrition, cirrhosis and severe burns are well-recognized predisposing factors for the development of EPM (13). However, hypocortisolism as a predisposing factor for EPM has only recently been recognized (14). Although the rapid correction of hyponatremia of any aetiology can lead to EPM, chances increase in the setting of undiagnosed hypocortisolism. Rapid steroid replacement in the setting of hypocortisolism along with the correction of hyponatremia by 3% saline can add to damage due to a steep rise in sodium levels. The patient in the present study had a history of secondary amenorrhea and lactation failure; however, Sheehan's syndrome was not suspected at her initial medical contact. Thus, the case in the present study also reiterates the importance of a basic history and clinical examination, while treating an electrolyte imbalance. In general, demyelination is associated with a poor prognosis with the persistence of morbidity following treatment. However, EPM associated with hypocortisolism has been shown to have a more favourable outcome, as observed in the patient described herein (15).

In conclusion, the present study describes a case with the first-time presentation of Sheehan's syndrome as a myxedema crisis. Sheehan's syndrome remains prevalent in developing countries and should be suspected in females with a history of lactation failure and secondary amenorrhea especially with home delivery. The present case report reiterates the importance of basic history and clinical examination, while treating an electrolyte imbalance and exercising caution during correction. The timely suspicion of myxedema crisis in patients presenting with an altered sensorium in the emergency room remains the most crucial part of managing the condition. Furthermore, the case described herein highlights the importance of being vigilant during the recovery phase of a myxedema crisis, and considering alternative causes if altered sensorium persists.

## Figures and Tables

**Table I:** Hormonal profile of the patient in the present case report.

Parameter	Patient value	Reference range
T3	<40 ng/dl	70-204 ng/dl
T4	3.04 µg/dl	5-11 µg/dl
TSH	1.14 µIU/ml	0.550-4.780 µIU/ml
Prolactin	1.75 ng/dl	3.34-26.72 ng/ml
ACTH	5.9 pg/ml	7.2-63.3 pg/ml
Cortisol	3.95 µg/dl	4.3-22.4 µg/dl
LH	5.4 mIU/ml	1.9-12.5 mIU/ml
FSH	3.11 mIU/ml	3.85-8.78 mIU/ml
Estradiol	10 pg/ml	19.5-144 pg/ml

TSH, thyroid stimulating hormone; ACTH, adrenocorticotropic hormone; LH, luteinising hormone; FSH, follicle stimulating hormone.

## Data Availability

Data sharing is not applicable to this article, as no datasets were generated or analyzed during the current study.
